# Controlling the Resit Effect by Means of Investment Depreciation

**DOI:** 10.5334/joc.40

**Published:** 2018-07-16

**Authors:** Rob Nijenkamp, Mark R. Nieuwenstein, Ritske de Jong, Monicque M. Lorist

**Affiliations:** 1 Department of Experimental Psychology, University of Groningen, Groningen, NL; 2 Neuroimaging Center Groningen, University Medical Center Groningen, University of Groningen, Groningen, NL; 3 Research School of Behavioural and Cognitive Neurosciences, University of Groningen, Groningen, NL

**Keywords:** resit exams, investment decisions, investment depreciation, education, mathematical modeling, rationality

## Abstract

In accordance with a rational model of study-time investment, we previously found that the prospect of a resit exam leads to lower investments of fictional study-time for a first exam opportunity in an investment game utilizing simulated exams. In the current study, we investigated whether the depreciation of one’s first-exam investment reduces the resit effect. Specifically, we investigated study-time investments for a simulated multiple-choice exam in which 0, 50, or 100% of the initial study-time investment was lost before the resit exam. In accordance with our predictions, we found that the magnitude of the resit effect decreased as investment depreciation increased. This finding suggests that the negative effect of resit exams on study-time investment may be countered by creating conditions under which investment depreciation (i.e. forgetting) is expected to occur, for instance, by increasing the temporal interval between the first attempt and resit exam.

Research on decision making has generally focused on situations in which individuals are asked to make a single choice. Recently, however, two lines of investigation have provided evidence that choice behavior may change in interesting ways when the prospect of a second chance is introduced. In one line of research it was found that the availability of a second opportunity to achieve a goal may lead to a reduced probability of achieving this goal in a first opportunity to do so (Napolitano & Freund, 2016; see also Shin & Milkman, 2016; Napolitano & Freund, 2017). In a second line of work, studies on the effects of resit exams – the opportunity to re-do a failed exam in order to pass a course – suggest that study-time investment for a first exam may be substantially lower if a resit is available (Kooreman, 2013a; Nijenkamp, Nieuwenstein, De Jong, & Lorist, 2016; Michaelis & Schwanebeck, 2016). It is this latter phenomenon – the resit effect – that the present study is specifically concerned with.

As proposed by Kooreman (2013a) and empirically verified by Nijenkamp and colleagues (2016), the resit effect can be understood in terms of utility maximization. Specifically, the availability of a resit exam is assumed to change the tradeoff between the costs of study-time investment and the benefits of enhanced passing probability in such a way that rational students – that is, those motivated to optimize this tradeoff through maximizing expected utility – will now find an optimum at a reduced study time investment for the first opportunity. As pointed out by Kooreman (2013a), resit exams thus present a potential windfall gain for students, enabling them to gain, on average, an overall higher passing rate with a lower average total study-time investment. From a broader educational perspective, the effects of resit exams on students’ study strategies should be cause for concern, as this windfall gain would be completely due to students being provided an opportunity to pass at the first attempt with reduced study-time investment, and thus less knowledge, as compared to a no-resit policy. If, despite this reduced investment, a substantial proportion of students succeeds in passing at the first attempt, the result will be that the average level of knowledge across all students who passed the exam, either at the first or the second attempt, will be lower than that in case of a no-resit policy, potentially compromising the achievement of intended learning objectives.

A number of restrictive policies have been proposed to maintain the benefits and reduce the potential drawbacks of resit exams, including the windfall gain that is likely to materialize if resit opportunities are offered freely and unconditionally. For instance, we have found in a laboratory study (Nijenkamp et al., 2016) that the resit effect was markedly attenuated when access to the resit was made probabilistic (50%) as opposed to unconditional, whereas the effect was hardly affected when access was made conditional upon obtaining a minimum non-passing grade (4 on a 1–10 scale) on the first exam. Likewise, Michaelis and Schwanebeck (2016) discussed several examination policies that might help to attenuate the resit effect, such as imposing a financial charge for the resit, capping resit marks at the pass threshold, listing the number of attempts needed to pass in the exam report, and malus points accounts.

While these suggestions may be relevant and useful in some educational settings, some are likely to raise multiple concerns regarding, for instance, equal opportunity and fairness. In the present study we focused on the potential for controlling the resit effect through a potentially less controversial factor or manipulation, *depreciation of initial investments* – that is, the extent to which the effort put into achieving one’s goal during a first attempt fails to yield any savings or benefits for the second attempt to achieve the same goal. This factor is particularly relevant, and probably unavoidable, when considering the case of resit exams, as the resit effect could well depend on the extent to which the student expects to benefit from any savings from the first learning episode towards the exam. Similarly, we could have focused on other factors that likely influence the resit effect, such as students capitalizing on the likelihood of the resit exam questions being similar in content to the first exam (see also Kooreman, 2013a; Michaelis & Schwanebeck, 2016), or the fact that the first exam functions as a learning opportunity to be better prepared for the resit (i.e., the testing effect; e.g., see Roediger & Karpicke, 2006). Since we aimed to provide a first empirical test of the effect of investment depreciation on the resit effect, however, and given that the above-mentioned effects currently cannot be readily implemented in our investment-game paradigm, we chose to solely focus on investment depreciation in the current study.

Depreciation of initially invested study time, conceptualized more commonly as *forgetting* of some of the knowledge that was acquired when studying for the first attempt, intuitively would seem to make a resit a less attractive option, as it would require students to spend extra study time to regain lost knowledge. However, Michaelis and Schwanebeck (2016) pointed out that forgetting could affect the resit effect in multiple, and sometimes opposite ways. For example, if one is already expecting to resit an exam it would not be useful to invest a high amount of time on the first exam in that case, as much of this effort will be in vain due to forgetting. On the other hand, and as is the case for our model outlined below, if one wants to optimize the utility of ones investment while being faced with forgetting, it is optimal to invest more on a first exam in order to avoid the resit. Regardless of the ambiguities surrounding the potential effect, forgetting is a pervasive property of human memory and the degree of forgetting is known to depend on the length of a retention interval (Ebbinghaus, 1885; see also Rubin & Wenzel, 1996). Therefore, manipulating the degree of forgetting by increasing the time between first exam opportunity and the resit exam might provide effective means to counteract the resit effect. Similarly, manipulating students’ beliefs about their own forgetting might already provide means to counteract the resit effect, as it has been shown that individuals tend to underestimate the amount of forgetting that will occur in between moments of learning and moments of testing (Koriat, Bjork, Sheffer, & Bar, 2004; Kornell & Bjork, 2009). The task used in the current paper, however, is modeled after a situation where the effects of forgetting are known and simply the degree of forgetting is manipulated.

Previously, we briefly described preliminary results of model simulations on possible mitigating effects of depreciation on the resit effect (Nijenkamp et al., 2016). In the next section, we present and explain these simulation results and model predictions in detail. Interestingly, while our model predicts proportionally large mitigating effects of depreciation on the resit effect, the original model of Kooreman (2013a; see also Kooreman, 2013b) predicts little or no such effects, whereas Michaelis and Schwanebeck (2016) state that their model simulations show overall mitigating effects of depreciation on the resit effect, but they provide no indication as to the size of these effects. We then report the results of a laboratory study designed to test the model’s predictions or, more precisely, to test people’s ability to conform to the model predictions and thus deal optimally, from a utility maximization perspective, with known degrees of depreciation of initial investments in a resit scenario.

## Modeling the Effects of Depreciation

**Figure 1:**
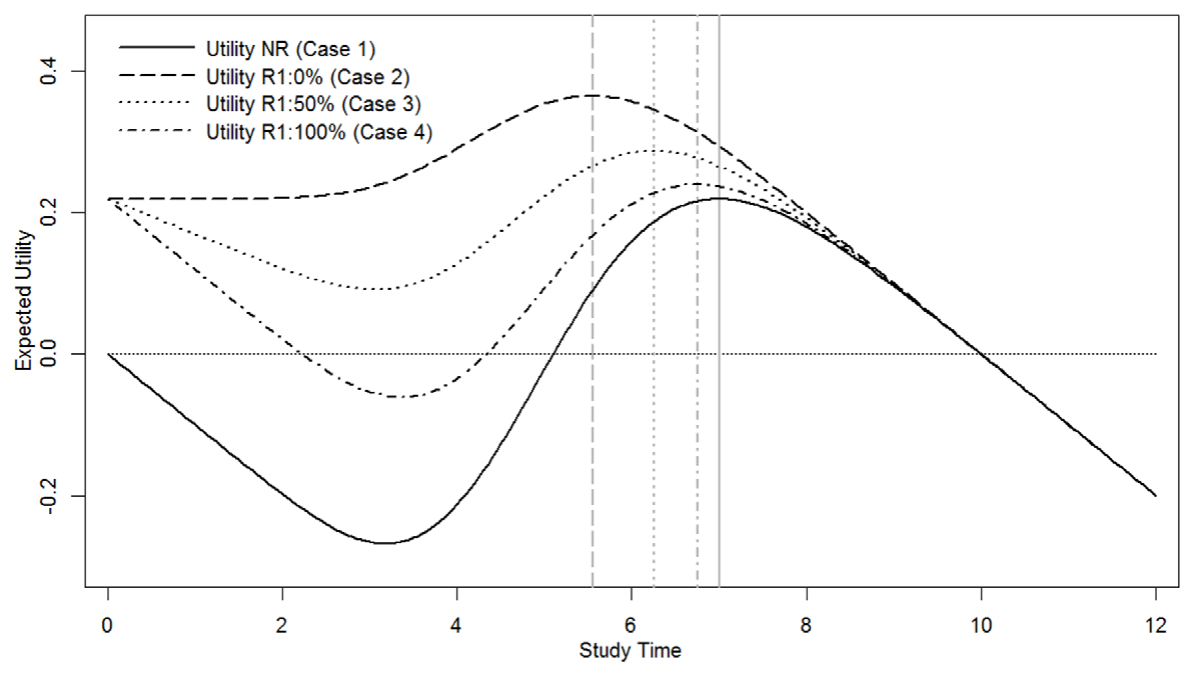
**Model predictions.** Model predictions for expected utility as a function of invested study time for the first exam, without a resit (NR; Case 1) or with a resit with no depreciation (R1:0%; Case 2), 50% depreciation (R1:50%; Case 3), or 100% depreciation (R1:100%; Case 4) of invested study time. The vertical gray lines represent the points of maximum utility in each condition.

The model of study-time investment presented and validated in our previous paper (Nijenkamp et al., 2016) builds upon Kooreman’s (2013a) model, but critically extends it by implementing an empirically well-established exponential learning function and by formalizing the probabilistic relationship between acquired knowledge and passing probability through the use of a fictional multiple-choice exam. The model assumes that students will seek to maximize expected utility associated with studying for and taking an exam, where expected utility is a sole function of invested study time for the first exam. In case of a single exam (no resit), we denote the study time at which expected utility peaks by *t_optimum_* (see the solid vertical gray line in Figure 1) and the associated passing probability by *p_optimum_*. The base model without forgetting (see Supplementary 2 for an overview) assumes that if there is a resit opportunity and the first exam has been failed *t_optimum_* does not change. Therefore, the effective total amount of study time invested for the resit, that is, the sum of invested study time for the first exam and any additional invested study time for the resit, will always be equal to the optimal time investment for a single exam opportunity (i.e. *t_optimum_*). In other words, if first-exam investments are less than *t_optimum_*, the amount of additional time invested on the resit will be to get the total time invested on both exams to equal *t_optimum_*; Additionally, if the time invested on the first exam is more than *t_optimum_*, no additional time will be invested on the resit – this is the reason that expected utility is a sole function of invested study time for the first exam also in case a resit opportunity is available. In the present study, we implemented the possibility of depreciation of study-time investment in the model by means of two minor extensions to its mathematical structure. Formula 6 from Nijenkamp et al. (2016) is extended with a parameter (1–D):

{t_{R2}} = \max \left({{t_{optimum}} - \left({1 - D} \right)^{*}{t_{R1}},\,\,0} \right)

With *t_R_*_2_ denoting the additional study-time investment for the resit exam (R2), *t_optimum_* representing the optimal time investment, *D* denoting the level of depreciation, with a value between 0 and 1, and *t*_*R*1_ denoting the study-time investment for the first exam (R1). Note that (1 – D)*t_*R*1_ denotes the effectively saved study-time investment for the first exam. Formula 7 from Nijenkamp et al. (2016) is similarly extended with parameter (1–D):

{p_{pass\,R2}} = max\left({{p_{optimum}},{p_{pass}}\left({\left({1 - D} \right)^{*}{t_{R1}}} \right)} \right)

With *p*_*pass R*2_ denoting the passing probability for R2, *p_optimum_* representing the optimal passing probability, and *p_pass_* denoting the passing probability for an exam as a function of a particular time investment (see Supplementary File 2 for a full overview of the model).

Predictions of the extended model are depicted in Figure 1. The figure shows expected utility as a function of invested study time for the first exam for four cases (in each case the utility associated with passing the exam was set to 1 and the cost per unit of invested study time set to 0.1 – see Supplementary File 2 for details):

**Table:** 

1.	Exam without resit opportunity (NR). As the investment of study time increases from 0, utility initially decreases because passing probability initially remains close to 0. As invested study time increases further, however, utility starts to increase as passing probability begins to rise quickly (see Figure 2), and eventually peaks at *t_optimum_*.
2.	Exam with resit opportunity and 0% depreciation. Utility starts at the maximum expected utility for the no-resit case – this is due to the fact that the model assumes an optimal investment of study time for the resit. At low levels of invested study time, utility remains at this constant level, which is jointly due to the fact that passing probability initially remains close to 0 and the fact that any invested study time on the first exam is fully transferred to the resit in the absence of depreciation. As invested study time increases further and passing probability quickly rises, expected utility reaches a maximum that is substantially higher than that in the no-resit case, at a time investment that is substantially lower than *t_optimum_*. This is the resit effect, aptly characterized by Kooreman as a windfall gain for rational students. These predictions of the model were empirically verified by Nijenkamp and colleagues (2016) in a laboratory study.
3&4.	Exam with resit opportunity and 50% or 100% depreciation. These two cases are of central interest in the present study. In contrast to the utility function for the resit scenario with 0% depreciation, the functions for these two cases show a decrease of expected utility across the initial range of invested study time for which passing probability remains close to 0 – this reflects the partial (50%) or complete (100%) lack of transfer of invested study time to the resit. Expected utility subsequently rises as passing probability quickly rises for larger time investments, and it can be seen to reach maxima at time investments smaller than *t_optimum_*. However, both in terms of the reduction of optimal study-time investment and of enhanced maximum expected utility, the resit effect can be seen to be reduced by about 45% and 85%, for 50% and 100% depreciation respectively, as compared to the effect obtained for 0% depreciation (see the vertical gray lines in Figure 1). This suggests that anticipated forgetting, and, as discussed later, various ways of manipulating the degree of such anticipated forgetting, may offer great potential for mitigating unwanted resit effects.^1^

**Figure 2:**
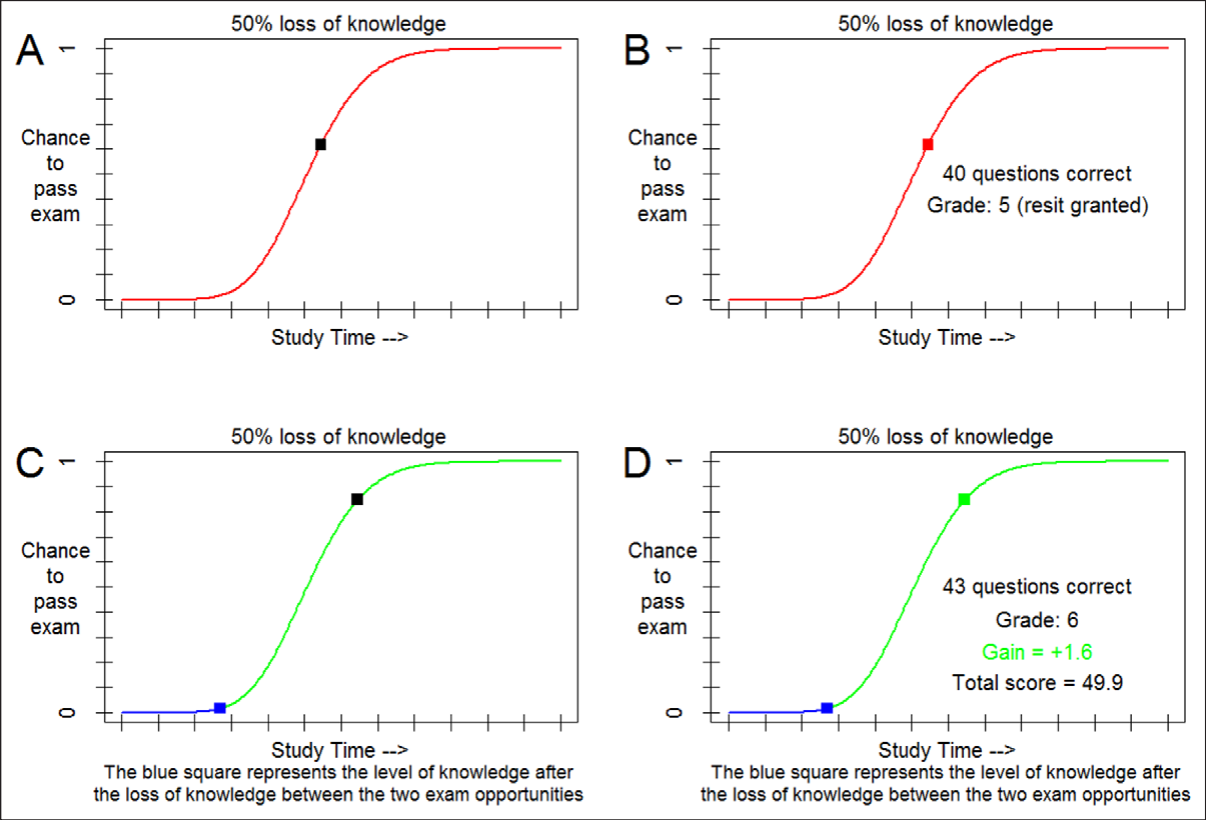
**Example trial.** The plot that was used as the stimulus material, showing the relationship between study-time investment (x-axis) and the probability of passing a simulated exam (y-axis). Illustrated here is a trial from the 50% depreciation condition in which the first exam is failed. Panel A illustrates the moment at which participants make their R1 investment at the point indicated by the cursor. Panel B illustrates the end of the R1 investment, where access to the resit is granted. Feedback consisting of the number of correctly answered questions, the grade, and whether access to R2 is granted is given. After the feedback, participants see the blue line and square move from the position of the red square (the R1 investment) to the position on the curve that is equal to 50% of the R1 investment. Panel C illustrates the start of R2, with the blue line and square representing the depreciated R1 investment and the black square representing the selected R2 investment. Panel D illustrates the end of R2, with the green square indicating the final R2 investment. Feedback consisting of the number of correctly answered questions, the grade, the gains/losses, and the total earnings up until that point is given. Calculations for the gains/losses for R2 are based on the initial R1 investment and the additional time investment on R2, counted from the starting point after depreciation (i.e. the blue square).

In the present study, these four cases were used to test people’s ability to conform to the model’s predictions and thus deal optimally, from a utility-maximization perspective, with known degrees of depreciation of initial investments in specific resit scenario’s. The basic resit effect, obtained by contrasting the no-resit condition with the 0%-depreciation condition with resit, has been empirically obtained before (Nijenkamp et al., 2016), and we expect to replicate this finding here. Most important for the present purposes is the question whether depreciation will lead participants to adopt a more conservative study-time investment strategy, resulting in a reduction of the basic resit effect by approximately 45% and 85%, for 50% and 100% depreciation, respectively, as predicted by the model.

## Method

### Participants

Seventy first-year psychology students (17 male) from the University of Groningen participated in the experiment in return for course credit. Their age ranged from 17 to 28 (*M* = 19.9 years, *SD* = 2.03). Approval of the Ethics Committee Psychology was obtained before the start of data collection. Written informed consent was obtained from all participants prior to the experiment.

### Materials

The study-time investment task (Nijenkamp et al., 2016) was programmed in MATLAB and run on computers situated in a room with 5 computer set-ups, which were enclosed by paperboard walls. The stimulus for the task consisted of a graph which depicted the relationship between study time, defined in terms of 12 arbitrary units along the x-axis, and the probability of passing a simulated 60-item multiple-choice exam along the y-axis (Figure 2). The graph was the same on every trial, for every participant, and included a cursor that could be moved along the curve so as to allow the participant to select a desired amount of study time.

### Design and procedure

The study-time investment task (see Figure 2) consisted of a graph showing a curvilinear function relating study-time investment to the probability of passing a simulated 60-item multiple-choice exam. Participants were asked to indicate their choice of study-time investment for passing the simulated exam. They were informed that they would pass this exam if their grade was a 6 (out of 10) or higher. To select the desired amount of study time and associated passing probability, participants could move a cursor along the curve in the graph, and they had to click the mouse to select the amount of study time they wanted to invest. After their investment, feedback on the outcome of the simulated exam was generated by the model based on the amount of invested study time. This feedback was shown to the right of the curvilinear function.

The availability of a resit option was manipulated within-subjects in a blocked design, such that each participant completed 6 blocks of 60 trials, of which 3 blocks included a resit whereas the other blocks did not. The resit and no-resit conditions were presented in alternation, and half the participants started with a resit condition, whereas the other half started with a no-resit condition. On a between-subjects level it was manipulated how much of the invested time on R1 was depreciated (i.e. forgotten) when moving to R2 (0, 50, or 100%). Prior to starting the task, participants completed the CRT (Frederick, 2005), an index of rationality (Toplak, West, & Stanovich, 2011). As we previously found a positive correlation between the resit effect and CRT scores (Nijenkamp et al., 2016) participants were assigned to a depreciation condition based on their CRT score (0%-depreciation: *n* = 27, *M* = 1.56, *SD* = 1.15; 50%-depreciation: *n* = 22, *M* = 1.54, *SD* = 1.22; 100%-depreciation: *n* = 21, *M* = 1.52, *SD* = 1.25). In a similar fashion, as gender differences have been found with regards to CRT scores (Frederick, 2005), we attempted to arrive at an equal distribution of male and female participants across conditions by assigning participants to conditions on the basis of their gender (0%-depreciation: *n*_Male_ = 8, *n*_Female_ = 19; 50%-depreciation: *n*_Male_ = 4, *n*_Female_ = 18; 100%-depreciation: *n*_Male_ = 5, *n*_Female_ = 16).

Prior to starting the task, participants were informed about the nature of the exam, the required number of correct answers to pass the exam, the nature of the relationship between study-time investment and the probability of passing the exam, and how much of their R1-investment they would lose when moving to R2. In addition, participants were informed that each unit of study time would cost them 1 point whereas they would earn 10 points if they passed the exam. A failure to pass the exam resulted in a loss in points that was dependent on the amount of time they had chosen to invest. After deciding on their investment, participants were informed of the number of correct answers, the resulting grade, the number of lost or gained points, and they also received information about their total net earned points up to that moment in the task.

In the resit condition, participants moved on to the next trial if a passing grade was obtained on R1. If R1 yielded a failing grade, they moved on to R2 after the result of R1 had been presented for 2.5 seconds. In case participants lost 50% or 100% of their investment, they saw the cursor move to the position that corresponded to respective percentages of their R1-investment. In case of 0% loss, the choice of study-time investment for R2 was restricted to an amount equal or higher than the study time invested for R1. This was made clear by presenting the cursor on the study time invested for R1, or at the respective point after depreciation, and by coloring the curve above this point green, thus highlighting which amount of time could be invested for R2. To indicate the knowledge savings in case of depreciation the part of the curve below the new starting point was colored blue (see Figure 2). Participants received information on the meaning of part of the curve turning blue.

### Data analysis

For the data analysis we used the JASP software package (Love et al., 2015). We computed Bayes factors to assess the extent to which the data provided evidence in favor or against our predictions (see Rouder, Speckman, Sun, Morey, & Iverson, 2009). As a first step we assessed the influence of our depreciation manipulation using a Bayesian one-way ANOVA. Next, we used Bayesian t-tests to assess whether the predicted resit effects, defined as the difference in study-time investments between NR and R1, were larger than zero, whether their magnitude was equal to the model’s predictions, and to compare the resit effects between the depreciation conditions. Furthermore, we used Bayesian ANOVAs and t-tests to compare the passing probability and average total study-time investment per passed exam across conditions. For the resit conditions, the total study time was computed as the sum of time invested for R1 and the additional time that was invested for R2 (after depreciation of the R1 investment).

In reporting the results of the Bayes factors analyses we adhered to Wetzels et al. (2011) in classifying *BFs* ≥ 3 and ≤ 10 or ≥ .1 and ≤ .33 as “substantial” evidence in favor of *H*_1_ or *H*_0_, respectively, whereas *BFs* between 10 and 30 or between .03 and .1 were classified as “strong” evidence, and *BFs* between 30 and 100 or between .01 and .03 were classified as “very strong” evidence, and *BFs* > 100 or < .01 were classified as “decisive” evidence. Additionally, we classified *BFs* between 1 and 3 or between 0.33 and 1 as ‘anecdotal’ evidence in favor of *H*_1_ or *H*_0_, respectively.

### Outlier exclusion

Investments of less than 2 or more than 10 study-time units were classified as outliers and excluded from further analysis. This entailed a loss of 1.3% of all recorded trials. Comparing the results with and without the outliers, it was found that the exclusion of these outliers did not affect the results.

## Results

### Depreciation and the resit effect

**Figure 3:**
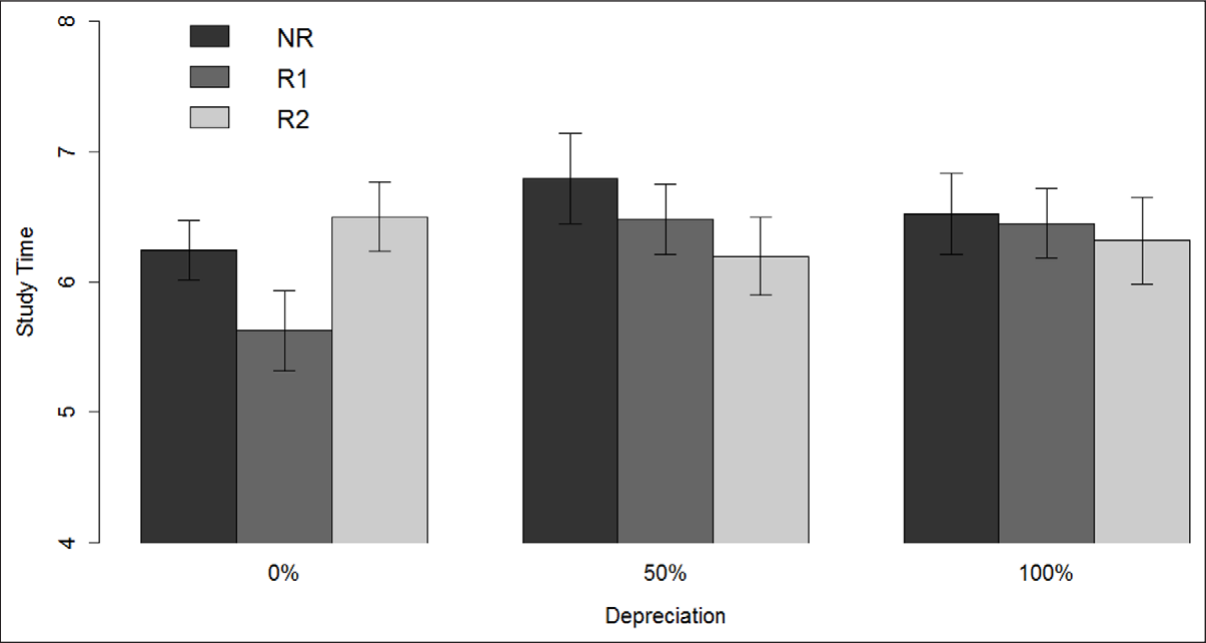
**Mean study-time investments.** Mean study-time investments for NR (single exam), R1 (first exam with resit opportunity), and R2 (resit exam) per depreciation condition (0%, 50%, or 100%). With depreciation, the total R2 time presented corresponds to the objective R2 investment (i.e. the R2 investment without the addition of the depreciated R1 investment).

A Bayesian ANOVA on the mean difference in time invested between NR and R1 (i.e. the resit effect) for each participant revealed that there was strong evidence in favor of an effect of depreciation (*BF*_10_ = 19.35), with the resit effect being smaller for conditions with greater degrees of depreciation (see Figure 3). Specifically, follow-up *t*-tests showed that there was very strong evidence (*BF*_10_ = 77) that the magnitude of the resit effect was larger with 0% depreciation than with 100% depreciation, but there was only anecdotal evidence that the resit effect was larger with 0% than with 50% depreciation (*BF*_10_ = 2.2), and with 50% than with 100% depreciation (*BF*_10_ = 2.2).

Next, we examined whether each of the three depreciation conditions showed a reliable resit effect, as predicted by the model. Replicating our previous findings (Nijenkamp et al., 2016), there was decisive evidence (*BF*_10_ = 1686) in favor of a resit effect in the condition with 0% depreciation, meaning that less time was invested on R1 than on NR (Figure 3). Likewise, we found strong evidence (*BF*_10_ = 12.3) in favor of a resit effect in the condition with 50% depreciation. However, for the condition with 100% depreciation, there was only anecdotal evidence (*BF*_10_ = 0.6) for a resit effect.

In a subsequent series of analyses, we compared the magnitudes of the observed effects to the magnitudes of the effects predicted by the model. For the conditions with 0, 50, and 100% depreciation, the model predicted that the resit effect would be equal to a reduction of 1.4, 0.7, and 0.2 time units, respectively, whereas the observed mean resit effects equaled 0.6 (*SD* = 0.6), 0.3 (*SD* = 0.5), and 0.1 (*SD* = 0.3) time units, respectively, for these conditions. Bayesian tests comparing the observed to the predicted resit effects showed that there was decisive (*BF*_10_ = 3.16e^–5^) and very strong evidence (*BF*_10_ = 0.03) against the resit effect being equal to the model’s prediction for the 0% and 50% depreciation conditions, respectively, and no evidence (*BF*_10_ = 1.13) for either hypothesis for the 100% condition. Importantly, however, although the absolute magnitudes of the resit effects and the depreciation effect did not match the magnitudes predicted by the model, the relative decrease in the magnitude of the resit effect across 0, 50, and 100% depreciation did match the relative decrease predicted by the model, such that the resit effect decreased by approximately 45% and 85% across the 50% and 100% depreciation conditions, respectively.

### Comparison of time invested per passed exam

An important aspect of the resit effect is that the reduction in study time invested for R1 (compared to NR) may entail a windfall gain such that students may occasionally pass their exams with less study time. To determine how the depreciation manipulation influenced this windfall gain, we ran a Bayesian ANOVA on the mean difference (i.e. resit minus no resit) in the average total amount of study time invested per passed exam. A passed exam in the resit condition constitutes an exam that is passed with passing R1, or with passing R2 after a failed R1. As can be seen in Table 1, the average amount of study time invested per passed exam was lower in the resit condition than in the no-resit condition for the 0%-depreciation condition, such that participants invested 0.2 (*SD* = 0.4) study-time units less per passed exam in the resit condition. According to a Bayesian one-sided *t*-test, the data yielded strong evidence (*BF*_10_ = 15.1) that this difference was smaller than 0. Importantly, however, a comparison of this effect across depreciation conditions showed it was reversed when depreciation was present, with the ANOVA showing decisive evidence (*BF*_10_ = 6.0e^6^) for a main effect of a difference across depreciation conditions. Specifically, participants now invested more time per passed exam in the resit than in the no-resit conditions, with the differences being *M* = 0.2 (*SD* = 0.6), and *M* = 0.8 (*SD* = 0.5), respectively, for the 50 and 100% depreciation conditions. Further analyses showed that the difference was unreliable for the 50% depreciation condition, where we found anecdotal evidence against the difference score being unequal to zero (*BF*_10_ = 0.7). However, there was decisive evidence (*BF*_10_ = 45512) that participants invested more study time per passed exam for the resit exams in the 100% depreciation condition.

**Table 1:** Mean study-time investment and standard deviation per passed exam per depreciation condition.

Depreciation	Condition	Total Study Time per Passed Exam		Percentage of Passed Exams	

		Mean (*SD*)	Mean Difference (*SD*)	Mean (*SD*)	Mean Difference (*SD*)

**D = 0%**	**No Resit**	6.3 (.6)	–0.2 (.4)	78.2 (11.7)	13.3 (7.7)
	**Resit**	6.1 (.6)		91.4 (6.4)	
**D = 50%**	**No Resit**	6.9 (.8)	0.2 (.6)	84.5 (9.3)	9.5 (7.5)
	**Resit**	7.1 (.4)		94.0 (4.9)	
**D = 100%**	**No Resit**	6.6 (.7)	0.8 (.5)	81.5 (11.8)	12.4 (7.4)
	**Resit**	7.4 (.4)		93.9 (5.9)	

*Note.* A passed exam in the resit condition constitutes an exam that is passed with passing R1, or with passing R2 after a failed R1. In the resit condition without investment depreciation study-time investments per passed exam are defined as the investment on R1, plus the additional investment on R2. In the resit conditions with depreciation, the total time per passed exam is defined as the R1 investment, plus the difference between the depreciated R1 investment and the R2 investment. D indicates the level of depreciation. The mean difference score reported is obtained after subtracting the mean of the no resit condition from the resit condition in each depreciation condition (i.e. a higher difference score corresponds to more time being spent per passed exam or a higher proportion of passed exams in the resit condition than in the no resit condition).

### Comparison of passing probability

Lastly, we examined how the depreciation manipulation influenced the percentage of passed exams in the resit and no-resit conditions. For these analyses, we compared the differences in the percentages of passed exams between the resit and no resit conditions across the three depreciation conditions. As can be seen in Table 1, all depreciation conditions yielded a higher passing probability for the resit exam than for the first exam opportunity, with the differences being *M* = 13.3 (*SD* = 7.7), *M* = 9.5 (*SD* = 7.5), and *M* = 12.4 (*SD* = 7.4) for the 0, 50, and 100% depreciation conditions, respectively. For each depreciation condition, the Bayesian analyses indeed showed that there was decisive evidence that the percentage of passed exams was higher for the resit than for the first exam opportunity, all *BF*_10_’s > 3366, and a Bayesian ANOVA revealed there was only anecdotal evidence for an effect of depreciation the difference between the depreciation conditions.

## Discussion

In the current study, we aimed to assess whether the resit effect (Nijenkamp et al., 2016) is counteracted by introducing depreciation of the study-time invested for a first exam opportunity. In accordance with our predictions, the results showed that participants invested less study time for an exam when a resit was present, and this resit effect decreased as the degree of depreciation increased. In other words, participants made a rational optimization of the trade-off between the costs of investing time and the benefits of passing the exam, depending on how much of their initial investment was depreciated when moving to the resit. Furthermore, although the model over-predicted the magnitude of the resit effect and the depreciation effect when considering the absolute outcomes, the degree to which the resit effect was reduced did match the relative decrease predicted by the model, such that the resit effect was decreased by about 45% and 85% for the conditions with 50 and 100% depreciation. Importantly, the occurrence of depreciation not only mitigated the resit effect on study-time investment, but it also countered the windfall gain that arises from the resit effect. Specifically, whereas the 0% depreciation condition showed that participants invested less time per passed exam in the resit condition, this effect was reversed when depreciation was present, such that these conditions showed that participants invested more, instead of less, time per passed exam in these resit conditions. Taken together, these findings suggest that conditions that foster the depreciation of study-time investments could be a potent means to counter the resit effect and the associated windfall gain of passing a first exam opportunity with a reduced amount of study time.

## Implications of the Results

Before discussing the implications of the results, it is important to restate that the conclusions reached in the current paper are based on data obtained from a laboratory task that required the investment of fictional study time to pass a simulated exam. As this task is an abstraction of real exam-taking, the following discussion should be taken as provisional.

Given the results of the current study, what can we say, by extension, about the implications of these results for resit policies? As mentioned in the introduction, investment depreciation between R1 and R2 can be seen as an analogue for the forgetting of knowledge over time between a first exam and a resit exam. Imagine the investment of study time on R1 as having a certain value or worth, and when this exam is not passed the value of the invested study time starts to decrease as more of the acquired knowledge from the study-phase for R1 is forgotten. The more time there is between a failed R1 and the R2, the more knowledge the students would have forgotten (Ebbinghaus, 1885; see also Rubin & Wenzel, 1996). Usually, students know when R2 is scheduled, theoretically allowing them to take this knowledge into account when investing study time for R1. This is supported by the finding that students adjust their study behavior to the way their curriculum is organized. For example it has been found that students’ study behavior changes when exams are more spread out over the year or when resit exams are less spread out over the year, with the result that overall study progress increased (Jansen, 2004; see also Crombag, van der Drift, & Vos, 1985; Van der Drift & Vos, 1987).

Therefore, to prevent the windfall gain that exists by allowing students unrestricted resit exams the scheduling of R2 should be such that it allows for the loss of knowledge to incur sufficient costs and to be a sufficient prospective deterrent for students. Given the relationship between forgetting and the passing of time, our results suggest that R2 should be scheduled as far away from R1 as possible for the resit to provide students with an extra chance of passing the exam, while abolishing the reduction of study-time investment per passed exam on R1. This recommendation is in contrast to publications that have advocated, although seemingly without empirical basis, that R2 should be scheduled closer in time to R1, so that students might capitalize on the already-acquired knowledge after learning for the first exam opportunity, supposedly increasing the student’s learning efficiency (e.g., Wijnen et al., 1992; Hulst & Jansen, 2002; Jansen, 2004). Our results suggest that such resit policies promote the use of resit exams, in the sense that students could reduce their study-time investment for R1 and capitalize on the windfall gain outlined in the introduction. Our recommendation does find support from Walsh (2010), who states that ‘fast resit exams’ do not allow for a period of serious remediation on the student’s part and allow students to capitalize on chance, and from Bruijns and Kok (2014), who state that resit exams scheduled close in time to the first exam opportunity might result in more students preparing insufficiently for a first exam opportunity. Additionally, our recommendation finds support from the finding that more students pass R1 if R2 is scheduled less favorably (Cohen-Schotanus, 2012), thus highlighting the potential to increase educational efficiency.^2^

Research on judgments of learning has shown, however, that individuals generally over- or underestimate how much knowledge they will have retained when they will make a test on the learned materials (for a review, see Koriat, 2007). Moreover, when taking the factor of time between learning and testing into account, individuals have generally been shown to have an indifference to the anticipated retention interval when judging how well their eventual performance will be, thereby not taking into account that forgetting will take place (e.g., Carroll, Nelson, & Kirwan, 1997; Maki & Swett, 1987; Shaddock & Carroll, 1997). Additionally, it has been shown that individuals underestimate the amount of forgetting that will occur in between moments of learning and moments of testing (Koriat, Bjork, Sheffer, & Bar, 2004; Kornell & Bjork, 2009). Despite the fact that individuals do not seem to possess accurate metacognitive perception of the amount of knowledge they will retain in the moments between learning and testing, however, participants did take depreciation into account when making fictional study-time investments in the current study, potentially highlighting fundamental differences between our resit paradigm and real-life study situations. Despite these potential fundamental differences, it is important to note that the relevance of using investment depreciation/forgetting to minimize the resit effect does not lie solely in scheduling R2 exams later in time, but also lies in training students’ metacognitive skills with regard to learning and forgetting. Future research should focus on bridging the gap between our resit paradigm and real-life study situations, and assess the existence of the resit effect and the effects of investment depreciation in real-life study scenarios. Furthermore, future directions for research should also include focusing on whether the availability of resit exams, and by extension forgetting, influences established memory effects such as, for example, the difference in retention of learned information through deep versus shallow learning (e.g., see Chin & Brown, 2000), massed versus spaced learning (e.g., see Kornell & Bjork, 2008), and the testing effect (e.g., see Roediger & Karpicke, 2006).

In the broader context of the recent literature on second chances and backup plans, that we referred to at the start of the introduction, a resit exam would seem to provide a particularly simple and clear example of a backup plan or, more precisely, a contingent backup plan, defined by Napolitano and Freund (2016) as a backup plan that links future actions to specific contingency conditions associated with failure of first-choice plans. Whereas such backup plans typically are intentionally developed and then held in reserve, which may introduce practical advance costs and detract resources from developing and using the first-choice plan (Napolitano & Freund, 2016; for exceptions, see Shin & Milkman, 2016), resit exams require no such advance investments from students. This greatly simplifies the development of formal models to compare different strategies in terms of associated expected utilities, as we did in the current paper. Interestingly, very similar to the resit effect reported here, recent research suggests that backup plans may lead to a reduced probability of achieving one’s goal by the first-choice plan (Napolitano & Freund, 2017; Shin & Milkman, 2016). As for the resit effect (e.g., Grabe, 1994), such effects of backup plans have been ascribed, at least in part, to negative effects on people’s motivation and effort to pursue the goal through Plan A (Napolitano & Freund, 2016, 2017; Shin & Milkman, 2016). As suggested by Shin and Milkman (2016), such effects may be mediated by a decreased desire to attain the primary goal. Our model suggests a quite different account, however, as it shows how negative effects of a second chance or backup plan on the effort invested in the first chance, or first-choice plan, need not reflect decreased motivation to achieve the goal. Rather, it might reflect a shift in the maximum of the curve relating invested effort in the first attempt and overall expected utility towards lower levels of effort (e.g., see Figure 1). In the present work, we investigated how such effects are modulated by the level of investment depreciation. This depreciation factor can be generalized to the question of the extent to which resources and effort invested in pursuing a goal in the first attempt, or with the first-choice plan, might benefit performance success in the second attempt, or backup plan. This factor appears to have received little or no attention in the literature on the effects of backup plans so far, with all of the studies in this field that we know of using virtually complete depreciation, or lack of transfer, from the first-choice plan to the backup plan (Napolitano & Freund, 2016, 2017; Shin & Milkman, 2016). The strong modulation by the level of depreciation of the resit effect found in the present study, suggests that potentially related effects of backup plans may be similarly modulated by degree and type of transfer between first-choice and backup plans.

## Additional Files

The additional files for this article can be found as follows:

10.5334/joc.40.s1 Supplementary file 1. Formal proof of resit effect with 100% depreciation.

10.5334/joc.40.s1 Supplementary file 2. Model as presented in Nijenkamp et al., 2016.

## Data Availability

Data can be accessed at https://doi.org/10.17605/osf.io/a9m7b.

## Footnotes

Note that with a depreciation factor of 100% (i.e. full forgetting) the model still predicts a small resit effect. This prediction is not tied to idiosyncrasies of the model, as it is easy to show that the derivative of the expected-utility function in this condition is negative at *t_optimum_* under very general assumptions. See supplementary file 1 for a formal proof.

Using a Bayesian ANOVA, we indeed found decisive evidence (*BF*_10_ = 860) for an effect of depreciation on the proportion of passed R1 exams in the resit condition. Compared to 0% depreciation (*M* = 62.5, *SD* = 18.8), we found very strong evidence for pass rates to be different in the 50% (*M* = 79.3, *SD* = 10.1; *BF*_10_ = 63) and 100% (*M* = 80.4, *SD* = 10.8; *BF*_10_ = 82) depreciation conditions. We found substantial evidence (*BF*_10_ = 0.3) against a difference in the proportion of passed R1 exams between 50% and 100% depreciation. Thus, our data supports the notion that a less favorable scheduling or R2 – meaning R2 is scheduled further away in time from R1 (i.e. more investment depreciation) – increases the proportion of passed R1 exams.

## References

[1] Bruijns, V., & Kok, M. (2014). Guidelines for Testing and Assessment.

[2] Carroll, M., Nelson, T. O., & Kirwan, A. (1997). Tradeoff of semantic relatedness and degree of overlearning: Differential effects on metamemory and on long-term retention. Acta Psychologica, 95(3), 239–253. DOI: 10.1016/S0001-6918(96)00040-6 9112803

[3] Chin, C., & Brown, D. E. (2000). Learning in Science: A Comparison of Deep and Surface Approaches. Journal of Research in Science Teaching, 37(2), 109–138. DOI: 10.1002/(SICI)1098-2736(200002)37:2<109::AID-TEA3>3.0.CO;2-7

[4] Cohen-Schotanus, J. (2012). De invloed van het toetsprogramma op studiedoorstroom en studierendement In: van Berkel, H., Jansen, E., & Bax, A. (eds.), Studiesucces bevorderen: het kan en is niet moeilijk. Bewezen rendementsverbeteringen in het hoger onderwijs, 65–78. Den Haag: Boom Lemma Retrieved from: https://www.boomlemma.nl/kernproduct/397/Studiesucces-bevorderen-het-kan-en-is-niet-moeilijk.

[5] Crombag, H. F. M., van der Drift, K. D. J. M., & Vos, P. (1985). De inrichting van curricula en het werkgedrag van studenten. Universiteit En Hogeschool, 31(5), 234–247.

[6] Ebbinghaus, H. (1885). Über das Gedächtnis Leipzig: Dunker.

[7] Frederick, S. (2005). Cognitive Reflection and Decision Making. Journal of Economic Perspectives, 19(4), 25–42. DOI: 10.1257/089533005775196732

[8] Grabe, M. (1994). Motivational deficiencies when multiple examinations are allowed. Contemporary Educational Psychology, 19, 45–52. DOI: 10.1006/ceps.1994.1005

[9] Jansen, E. P. W. A. (2004). The influence of the curriculum organization on study progress in higher education. Higher Education, 47(4), 411–435. DOI: 10.1023/B:HIGH.0000020868.39084.21

[10] Kooreman, P. (2013a). Rational students and resit exams. Economics Letters, 118(1), 213–215. DOI: 10.1016/j.econlet.2012.10.015

[11] Kooreman, P. (2013b). Corrigendum to “Rational students and resit exams” [Econom. Lett. 118 (1) (2013) 213–215]. Economics Letters, 121(1), 141–142. DOI: 10.1016/j.econlet.2013.07.005

[12] Koriat, A. (2007). Metacognition and consciousness. The Cambridge Handbook of Consciousness, 3(2), 289–326. DOI: 10.1017/CBO9780511816789.012

[13] Koriat, A., Bjork, R. A., Sheffer, L., & Bar, S. K. (2004). Predicting One’s Own Forgetting: The Role of Experience-Based and Theory-Based Processes. Journal of Experimental Psychology: General, 133(4), 643–656. DOI: 10.1037/0096-3445.133.4.643 15584811

[14] Kornell, N., & Bjork, R. A. (2008). Learning Concepts and Categories Is Spacing the ‘“Enemy of Induction”’?, 19(6), 585–592. 10.1111/j.1467-9280.2008.02127.x 18578849

[15] Kornell, N., & Bjork, R. A. (2009). A stability bias in human memory: Overestimating remembering and underestimating learning. Journal of Experimental Psychology. General, 138(4), 449–468. DOI: 10.1037/a0017350 19883130

[16] Love, J., Selker, R., Marsman, M., Jamil, T., Dropmann, D., Verhagen, A. J., Wagenmakers, E.-J., et al. (2015). JASP.

[17] Maki, R. H., & Swett, S. (1987). Metamemory for narrative text. Memory & Cognition, 15(1), 72–83. DOI: 10.3758/BF03197713 3821492

[18] Michaelis, J., & Schwanebeck, B. (2016). Examination rules and student effort. Economics Letters, 145, 65–68. DOI: 10.1016/j.econlet.2016.05.019

[19] Napolitano, C. M., & Freund, A. M. (2016). On the Use and Usefulness of Backup Plans. Perspectives on Psychological Science, 11(1), 56–73. DOI: 10.1177/1745691615596991 26817726

[20] Napolitano, C. M., & Freund, A. M. (2017). First Evidence for “The Backup Plan Paradox.” Journal of Experimental Psychology: General, 146(8), 1189–1203. DOI: 10.1037/xge0000331 28627908

[21] Nijenkamp, R., Nieuwenstein, M. R., De Jong, R., & Lorist, M. M. (2016). Do resit exams promote lower investments of study time? Theory and data from a laboratory study. PLoS ONE, 11(10). DOI: 10.1371/journal.pone.0161708 PMC5053496 27711140

[22] Roediger, H. L., & Karpicke, J. D. (2006). Test-enhanced learning: Taking memory tests imporves long-term retention. Psychological Science, 17(3), 249–255. DOI: 10.1111/j.1467-9280.2006.01693.x 16507066

[23] Rouder, J. N., Speckman, P. L., Sun, D., Morey, R. D., & Iverson, G. (2009). Bayesian t tests for accepting and rejecting the null hypothesis. Psychonomic Bulletin & Review, 16(2), 225–237. DOI: 10.3758/PBR.16.2.225 19293088

[24] Rubin, D. C., & Wenzel, A. E. (1996). One hundred years of forgetting: A quantitative description of retention. Psychological Review, 103(4), 734–760. DOI: 10.1037/0033-295X.103.4.734

[25] Shaddock, A., & Carroll, M. (1997). Influences on metamemory judgements. Australian Journal of PsychologyJournal of Psychology, 49(1), 21–27. DOI: 10.1080/00049539708259846

[26] Shin, J., & Milkman, K. L. (2016). How backup plans can harm goal pursuit: The unexpected downside of being prepared for failure. Organizational Behavior and Human Decision Processes, 135, 1–9. DOI: 10.1016/j.obhdp.2016.04.003

[27] Toplak, M. E., West, R. F., & Stanovich, K. E. (2011). The Cognitive Reflection Test as a predictor of performance on heuristics-and-biases tasks. Memory & Cognition, 39(7), 1275–1289. DOI: 10.3758/s13421-011-0104-1 21541821

[28] Van der Drift, K. D. J., & Vos, P. (1987). Anatomie van een leeromgeving, een onderwijseconomische analyse van universitair onderwijs. Lisse: Swets & Zeitlinger.

[29] Van Der Hulst, M., & Jansen, E. (2002). Effects of Curriculum Organization on Study Progress in Engineering Studies. Higher Education, 43(4), 489–506. DOI: 10.1023/A:1015207706917

[30] Walsh, K. (2010). Cost effectiveness in Medical Education. London: Radcliffe Publishing.

[31] Wetzels, R., Matzke, D., Lee, M. D., Rouder, J. N., Iverson, G. J., & Wagenmakers, E. (2011). Statistical Evidence in Experimental Psychology: An Empirical Comparison Using 855 t Tests. Perspectives on Psychological Science, 6(3), 291–298. DOI: 10.1177/1745691611406923 26168519

[32] Wijnen, W. H. F. W., Wolfhagen, H. A. P., Bie, D. D., Brouwer, O. G., Ruijter, C. T. A., & Vos, P. (1992). Te doen of niet te doen. Advies of de Studeerbaarheid van onderwijsprogramma’s in het hoger onderwijs. Den Haag: Ministerie van Onderwijs, Cultuur en Wetenschap.

