# Supplementary material for: Controlling the Resit Effect by Means of Investment Depreciation
Source: J Cogn. 2018 Jul 16;1(1):37. doi: 10.5334/joc.40 (PMC6634479; doi:10.5334/joc.40)
Supplement: Supplementary file 1. — Formal proof of resit effect with 100% depreciation. [file joc-1-1-40-s1.pdf]

Supplementary file 1: Formal proof of resit effect with 100% depreciation

In the current paper we presented an adaptation of our model of study-time investment on multiple-choice exams (Nijenkamp et al., 2016) that accommodates forgetting occurring between a (failed) first exam opportunity and a resit exam. For all levels of forgetting (i.e. 0%, 50%, and 100% depreciation) the model predicts a resit effect, that is the difference in invested time between a single exam and a first exam with resit opportunity. The fact that even for full forgetting our model predicts a resit effect might be surprising at first glance, however, a formal proof of the formulae shows that this predicted effect is simply the result of the mathematics of the model.

For a single exam opportunity, the utility as a function of invested study time is given by,

$$U(t_{NR}) = p(t_{NR}) - w \ast t_{NR},$$

and its derivative by,

$$\frac{dU}{dt_{NR}} = \frac{dp}{dt_{NR}} - w,$$

where this derivative is zero by definition at the optimal time investment ($t_{optimum}$),

For the case of a resit exam with 100% depreciation, the total utility as a function of invested study time for the first exam is given by,

$$U_{total} = U(t_{R1}) + \left(1 - p(t_{R1})\right) \ast U_{optimum},$$

and its derivative by,

$$\frac{dU_{total}}{dt_{R1}} = \frac{dU}{dt_{R1}} - \frac{dp}{dt_{R1}} \ast U_{optimum},$$

where $U_{optimum}$ is the maximum utility for a single exam (NR) associated with $t_{optimum}$. At t_{R1} = $t_{optimum}$, this derivative can easily be seen to assume a negative value, as the first term on the right

is zero by definition at $t_{R1} = t_{optimum}$ and, with $\frac{dp}{dt_{R1}}$ being strictly positive, the second term is strictly negative. This proofs that U_{total} must be maximal for some value t_{R1} that is smaller than $t_{optimum}$, even in the case of 100% depreciation.
